# Uncovering the Bioactive Potential of a Cyanobacterial Natural Products Library Aided by Untargeted Metabolomics

**DOI:** 10.3390/md19110633

**Published:** 2021-11-12

**Authors:** Leonor Ferreira, João Morais, Marco Preto, Raquel Silva, Ralph Urbatzka, Vitor Vasconcelos, Mariana Reis

**Affiliations:** 1 CIIMAR/CIMAR, Interdisciplinary Centre of Marine and Environmental Research, Terminal de Cruzeiros do Porto de Leixões, University of Porto, 4450-208 Matosinhos, Portugal; lferreira@ciimar.up.pt (L.F.); jmorais@ciimar.up.pt (J.M.); mpreto@ciimar.up.pt (M.P.); rssilva@ciimar.up.pt (R.S.); rurbatzka@ciimar.up.pt (R.U.); vmvascon@fc.up.pt (V.V.); 2 Departamento de Biologia, Faculdade de Ciências, Universidade do Porto, Rua do Campo Alegre, Edifício FC4, 4169-007 Porto, Portugal

**Keywords:** natural products library, cyanobacteria, cytotoxicity, 3D spheroids, untargeted metabolomics, MetaboAnalyst, GNPS

## Abstract

The Blue Biotechnology and Ecotoxicology Culture Collection (LEGE-CC) holds a vast number of cyanobacteria whose chemical richness is still largely unknown. To expedite its bioactivity screening we developed a natural products library. Sixty strains and four environmental samples were chromatographed, using a semiautomatic HPLC system, yielding 512 fractions that were tested for their cytotoxic activity against 2D and 3D models of human colon carcinoma (HCT 116), and non-cancerous cell line hCMEC/D3. Six fractions showed high cytotoxicity against 2D and 3D cell models (group A), and six other fractions were selected by their effects on 3D cells (group B). The metabolome of each group was organized and characterized using the MolNetEnhancer workflow, and its processing with MetaboAnalyst allowed discrimination of the mass features with the highest fold change, and thus the ones that might be bioactive. Of those, mass features without precedented identification were mostly found in group A, indicating seven possible novel bioactive molecules, alongside in silico putative annotation of five cytotoxic compounds. Manual dereplication of group B tentatively identified nine pheophytin and pheophorbide derivatives. Our approach enabled the selection of 7 out of 60 cyanobacterial strains for anticancer drug discovery, providing new data concerning the chemical composition of these cyanobacteria.

## 1. Introduction

Natural products continue to inspire many drug discovery programs; as such, more than sixty percent of the approved drugs comprise natural products, their synthetic derivatives, and their pharmacophore-inspired drugs [1]. Cyanobacteria have been regarded as one of the most promising groups of organisms capable of producing metabolites with pharmaceutical applications [2]. Since the 1970s, more than 1630 unique cyanobacterial compounds have been described [3], mainly belonging to the classes of non-ribosomal peptides (NRPs), ribosomally synthesized and post translationally-modified peptides (RiPPs), polyketides (PKs), and the hybrid NRPs/PKs [3,4]. These hybrid molecules contribute to the diversity of structural motifs found in cyanobacterial compounds. In addition, other classes of secondary metabolites have also been isolated from cyanobacteria as alkaloids, fatty acids, terpenes, and UV-protectant pigments [3,4]. Among the reported bioactivities, a great deal of studies have focused on the characterization of the cytotoxic and anticancer activity of cyanobacterial metabolites; among those, dolastatin 10, a tubulin polymerization inhibitor, is the most well-known [3,5]. Its synthetic derivatives monomethylauristatins yielded four approved antibody drug conjugates: Adcetris (2011) and Polivy (2019), used for the treatment of lymphoma; Padcev (2019), applied for the treatment of urinary tract cancers; and Blenrep (2020), for the treatment of relapsed and refractory multiple myeloma [6].

The Blue Biotechnology and Ecotoxicology Culture Collection (LEGE-CC, http://www.ciimar.up.pt/legecc/), hosted at CIIMAR (Matosinhos, Portugal), is a unique biological resource that hosts more than 700 strains of cyanobacteria, covering a wide range of geographical habitats. These are mainly represented by marine and freshwater water systems, but there are also representative strains from brackish, hypersaline, and terrestrial environments [7]. Regardless this biodiversity, this natural resource is still underexplored in terms of the discovery of new chemical entities. Our bioactivity-guided screening endeavors have delivered compounds such as lactylates of chlorinated fatty acids (chlorosphaerolactylates A–D) with antimicrobial effects [8]; chlorophyll derivatives with lipid reducing activity [9]; and compounds with anticancer activity, such as oxadiazine Nocuolin A [10], alkylresorcinol hierridin B [11], and the NRPs portoamides A–B [12] that also proved to have promising antifouling activity [13]. Despite these successes, our classic approach for cyanobacterial natural products discovery is often time consuming, originates false positives (synergistic effects), and ends frequently in the unsuccessful isolation of the active components due to their low concentrations. To overcome these problems and encompass the growing number of new cyanobacterial strains entering LEGE-CC, a new strategy for bioactivity screening is needed in order to accelerate our drug discovery process. In this study, we describe the generation of a natural products library and its assessment for potential cytotoxic activity. An untargeted metabolomics approach was used to discover and highlight the putative bioactive molecules.

## 2. Results and Discussion

### 2.1. Cyanobacterial Natural Products Library (LEGE-NPL)

**Figure 1:**
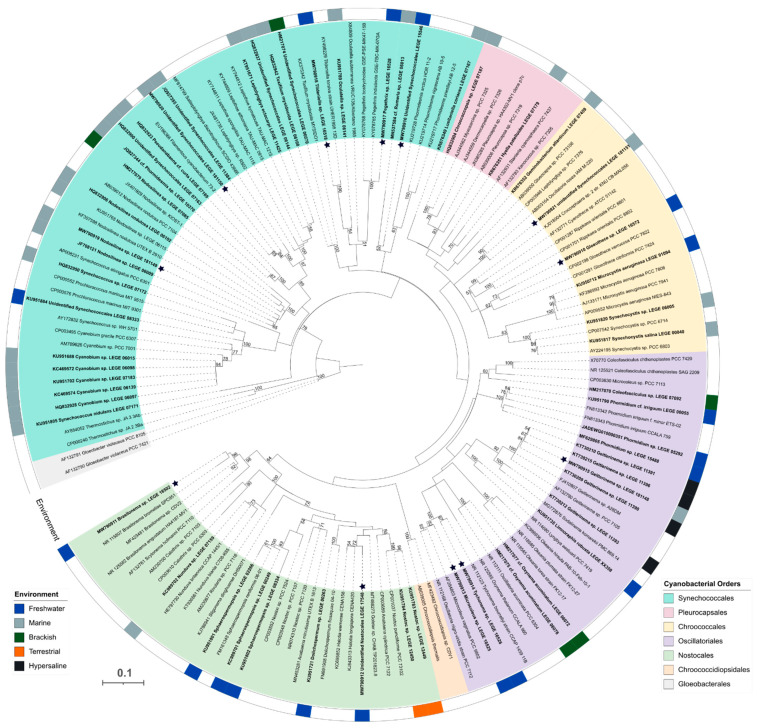
Maximum likelihood phylogenetic tree based on 146 partial 16S rRNA gene sequences of cyanobacteria. *Gloeobacter violaceus* PCC 7421 and *G. violaceus* PCC 8105 were used as outgroup. LEGE-CC strains used in this work are indicated in bold. The different color segments represent strain placement at order level following Komárek et al. [14]. Different colored strips around the tree represent the environment from where strains were isolated. Bootstrap values over 50% are indicated at the nodes. Black stars represent the strains whose sequences were obtained in this work.

In the last 10 years, LEGE-CC has had a significant increase in the number of deposited strains; nevertheless, its associated drug discovery has not been able to keep the same pace. As a possible solution for this problem, we designed a methodology for a cyanobacterial natural products library (LEGE-NPL). To test this approach, 60 cyanobacterial strains and 4 environmental samples were used (Table S1). The selected strains belong to different cyanobacterial orders following the classification of Komárek et al. [14]: Synechococcales (46%), Oscillatoriales (27%), Nostocales (15%), Chroococcales (10%), and Pleurocapsales (2%), representing the phylogenetic diversity of LEGE-CC (Figure 1). In addition, these orders have been considered to be a good asset for secondary metabolites research due to the richness in biosynthetic gene clusters found in their genomes [3,5,15].

The LEGE-NPL was designed to have a solid inventory (MeOH extracts) and a liquid inventory of fractions. The raw material that supplied the solid inventory was derived from 4 L cultures of cyanobacteria that yielded on average 2.57 g of dry weight (Figure S1). MeOH was chosen as solvent to produce the solid library because of its the ability to extract components with different polarities; previous results using sequential extraction did not show advantage of using different solvents over the single use of MeOH [9]. The average yield of extraction was 15.50% of the lyophilized biomass (Figure S1). The liquid inventory, constituted by eight fractions (denominated from A to H) derived from each MeOH extract, was designed to be fully compatible with a 96-well plate format for bioactivity screening. It was produced in semiautonomous fashion using a HPLC system coupled to an automatic injector, PDA detector, and an automatic fraction collector. Hence, the 64 MeOH extracts were separated on a C8 column using a gradient of H_2_O/MeCN, yielding a total of 512 fractions. The total run time, including gradient recovery, was 20 min per strain. These chromatographic conditions were optimized to ensure a good mass separation between all eight fractions that were estimated to have 2.50 mg. These plates were dried using a centrifugal evaporation system, resuspended in DMSO, and stored in 96-deep well plates as mother plates. The choice of the stationary phase considered the recent woks of the National Cancer Institute Program for Natural Products Discovery that indicated C8 as a preferred stationary-phase over the classical C18 or silica due to a better separation between lipophilic and medium polarity compounds [16].

### 2.2. Bioactivity Screening

Another aim of this work was to use cancer spheroids in routine screenings of LEGE-NPL. The cancer spheroids are characterized by a hypoxic core with quiescent cells and a prolific outer shell, and thus, they more accurately simulate the tumor microenvironment than 2D cultures. Due to this complexity, 3D culture systems are considered to be less prone to showing effects of unspecific activities or to overestimate the activity of compounds, increasing the chances of finding potent lead compounds [17].

The colon carcinoma cell line HCT 116 was chosen due to its ability to form uniform spheroids using the liquid-overlay technique, and because it had already been used in confirmation assays for cyanobacterial compounds [10,18]. Moreover, the assays using HCT 116 cells were used to compare the 2D versus 3D hit selection. The endpoints and readout techniques were adjusted accordingly to the nature of the cell culture system. For 2D cell cultures, cell viability was assessed by the standard MTT assay after a 48 h incubation period. This colorimetric assay was not suitable to measure cell viability in spheroids (Figure S2). This was verified mainly due to the poor diffusion of the dye, which can be attributed to the 3D matrices and tight cell–cell junctions present in the multilayer cell spheroids [19], resulting in low differentiation of the metabolic activity of the cells. Thus, for 3D cell cultures, cell viability was measured using the acid phosphatase assay after 96 h (longer exposure times in 3D cell cultures increase the sensitivity of the assay and reduce the false negative hits [19]). Moreover, to test the hypothesis that our methodology would be able to detect active compounds in fractionated extracts, the strain *Phormidium* sp. LEGE 05292 was included in the study set (as a positive control). This strain is known to produce the cytotoxic peptides portoamides A and B in a proportion of 3:1 [12]. This mixture presented IC_50_ values of 3.38 μM and 12.67 μM, respectively, to monolayer cultures and multicellular spheroids of HCT 116 cells. These results indicated that an approximate 4-fold higher concentration is needed to induce cytotoxicity in spheroids [18].

**Figure 2:**
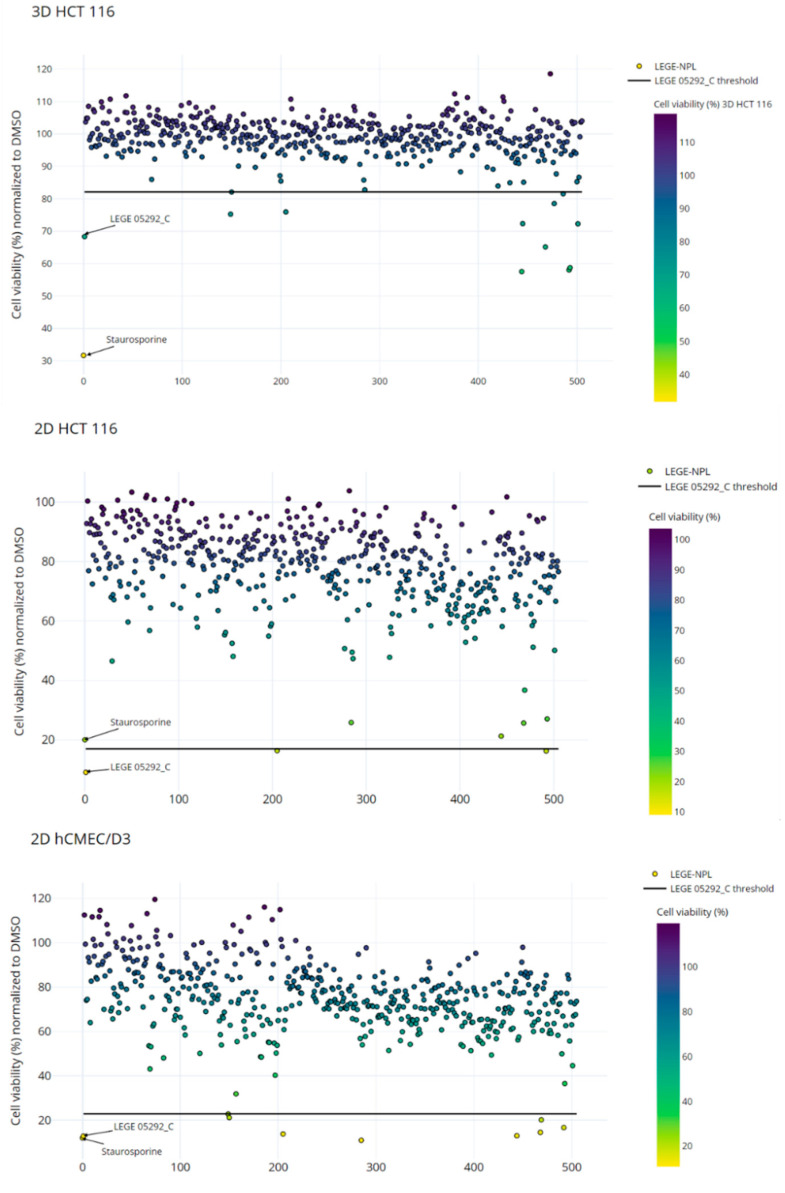
Cytotoxicity screening of 512 fractions of LEGE-NPL (at 25 µg mL^−1^) against 3D cell spheroids of HCT 116 cells, 2D HCT 116 cells, and hCMEC/D3. The percentage of cell viability was normalized to DMSO. The threshold for selection of positive hits was defined as mean viability of the cytotoxic faction LEGE 05292_C plus three times its standard deviation. All the assays were done in triplicate.

**Figure 3:**
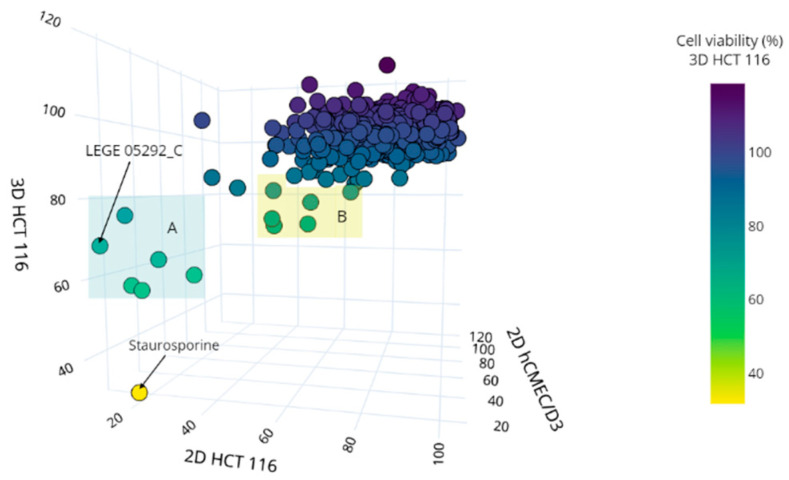
3D plot correlation of the cytotoxic profile of 512 fractions on the different cell models (3D HCT 116, 2D HCT 116, and hCMEC/D3). The x, y, and z axis represent the percentage of cell viability (normalized to DMSO). Two groups were defined based on the cytotoxic profile: group A and group B.

The 512 fractions of LEGE-NPL (25 µg mL^−1^) were screened for their cytotoxic effect on the colon carcinoma cells (2D HCT 116, 3D HCT 116) and a non-carcinogenic cell line hCMEC/D3 (Figure 2). The non-carcinogenic cell line was not used to select hits; instead it was used to test if the fractions exerted a generalized cytotoxicity or if they had selectivity towards cancer cells. The results were expressed as the percentage of cell viability normalized to the solvent control. To characterize the dynamic range of the assays, the Z’ factor was calculated using the positive (LEGE 05292_C) and solvent control (DMSO) data. The Z’ scores of 0.64–0.83 indicated that the mean and standard deviation of the controls were well separated [20], and thus the criteria to select positive hits was established as the mean viability of LEGE 05292_C plus three times its standard deviation ($\mu_{LEGE\;05292_{C}}+3\sigma_{LEGE\;05292_{C}})$. The monolayer assay with HCT 116 cancer cells had a hit rate of 0.4%, selecting the active fractions LEGE 181150_D and LEGE 17548_C (Figure 2). For the 3D HCT 116 cell assay, 11 active fractions were selected (2.1% hit rate) that correspond to one environmental sample and eight cyanobacterial strains (Figure 2). Contrary to what we expected, a higher hit rate was observed for the 3D spheroids than for the monolayer counterpart. Hence, the cell viability data from the three cell models was correlated in a 3D scatter plot to disclose any bioactivity tendency. As such, two bioactive groups could be recognized (Figure 3). Group A contains 5 fractions that present strong cytotoxicity towards the cancer and non-cancer cells (Table 1), whereas group B contains fractions selected for their activity in HCT 116 spheroids despite the moderate activity in the other monolayer assays. In light of these results, the fractions from both groups were selected for metabolomics studies in order to discover the potential cytotoxic compounds.

### 2.3. Group A: Metabolomics Analysis and Dereplication of the Putative Active Molecules

In an attempt to discover which metabolites could be responsible for the observed activity, an untargeted metabolomics analysis was performed. The metabolomes of fractions of group A were compared with a group of 12 fractions without activity on cancer spheroids (group C; Table 1). The extracted mass features with MZmine 2 were submitted to fold change (FC) analysis in MetaboAnalyst 5.0, which allowed for the potential differences in the metabolite profiles to be identified, and hence the bioactive compounds could be highlighted. 

**Figure 4:**
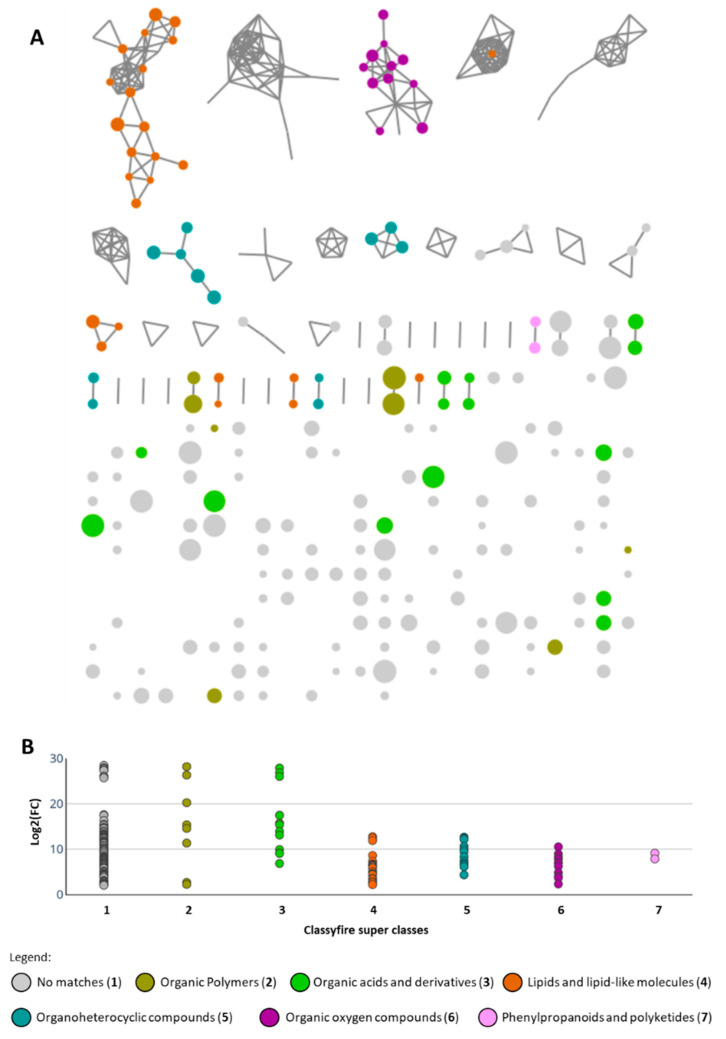
Feature-based molecular network of groups A/C annotated with MolNetEnhancer workflow. The nodes are color-coded accordingly to the ClassyFire super class classification, and their size is related to their fold change log2(FC) value (**A**). Distribution of the nodes with log2(FC) higher than 2 through each ClassyFire super class (**B**).

The chemical space of A/C was then represented as a molecular network constructed using the feature-based molecular networking workflow [21]. The characterization of the molecular families and annotation of compounds were estimated based on the integration of the in silico tools available from the Global Natural Product Social Molecular Networking (GNPS) platform: DEREPLICATOR [22], MS2LDA [23], Network Annotation Propagation (NAP) [24], and MolNetEnhancer [25]. The size of the nodes in the molecular networks was represented relative to the log2(FC) (Figure 4A).

The analysis of the molecular network revealed 191 nodes with log2(FC) between 2.00 and 28.45, of which 72 nodes were characterized as 13% lipids and lipid-like molecules, 8% organic acids and derivatives, 6% organic oxygen compounds, 6% organoheterocyclic compounds, 4% organic polymers, and 1% phenylpropanoids and polyketides, according to the ClassyFire super class classification [26]. The 17 top features with highest fold change (≥20) were distributed between the super classes’ organic polymers, organic acids and derivatives, and the category of no matches (Figure 4B). Such high fold change values reflect the uniqueness of these mass features among the studied metabolomes, which were found for all the active fractions except for LEGE 15488_C (Table 2). Detailed examination of these 17 ions indicated the majority to be related to the cytotoxic compounds portoamides A, B, and C, known to be produced by *Phormidium* sp. LEGE 05292 (Table 2) [27].

Moreover, in the fraction C of the unidentified Nostocales LEGE 17548 the cyclic lipopeptide minutissamide A was putatively annotated, together with an ion at *m*/*z* 1154.6172 [M+Na]^+^, that could correspond to a methylated minutissamide A (+14.01 Da mass shift). Minutissamide A was previously isolated from cultures of *Anabaena minutissima* (UTEX 1613), and its antiproliferative activity characterized using HT-29 human colon cancer cells (IC_50_ of 2.0 μM) [28], which correlates well with our bioactivity findings. However, *Anabaena minutissima* (UTEX 1613) and the unidentified Nostocales LEGE 17548 fall in different clades according to our phylogenetic study, the latter being more related to strains of the genus *Halotia* (Figure 1).

It is interesting to note that in the case of portoamides or minutissamides, the molecular network was not able to form clusters containing related ions. The absence of clustering led to poor propagation of library annotation as was observed for the sodium or potassium adducts of portoamides and minutissamides that were classified as “no matches”.

**Figure 5:**
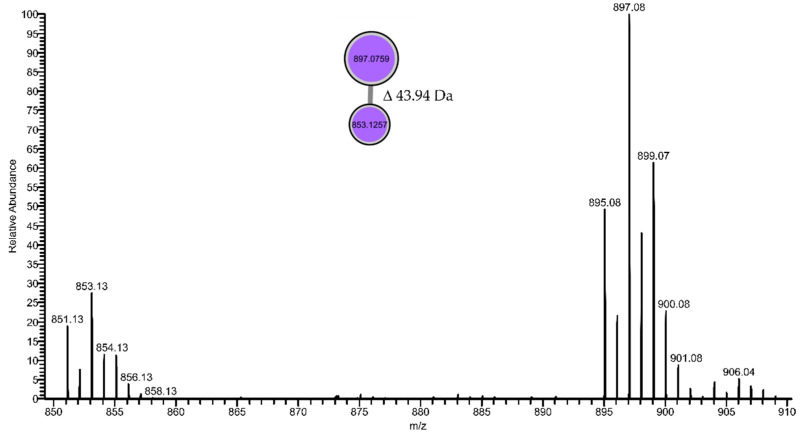
Mass spectrum and the respective cluster of two halogenated compounds present in fraction D of the unidentified Synechococcales LEGE 181150. The nodes are color-coded accordingly to the identity of the fraction. These mass features were exclusively found in the unidentified Synechococcales LEGE 181150.

Furthermore, four mass features, with significant fold change, could not be classified or dereplicated using the GNPS in silico tools or manual search in the databases Dictionary of Natural Products and CyanoMetDB [29], making them potential targets for the isolation of novel active compounds. Of these, the mass feature 897.0759 found in fraction LEGE 181150_D (Table 2) formed a cluster with another ion at *m*/*z* 853.1257 (7.81 min; log2(FC) = 17.43); analysis of the mass spectrum showed that these masses were in fact M + 2 isotope peaks, thus revealing the presence of halogenated atoms in these molecules. The complexity of the isotopic pattern suggests a combination of chlorine and/or bromine atoms (Figure 5). In addition, the mass difference of 43.94 Da between the compounds might correspond to Cl ↔ Br change. Preliminary GNPS experiments led us to two PhD theses reporting leptochelin (formerly phormidamide) [30,31], a compound with *m*/*z* 895.0786 and whose mass spectrum and isotopic pattern are very similar to our findings (*m*/*z* 895.0778; Δ 0.8 mDa). Nevertheless, the structure of this compound seems to not be fully elucidated yet. According to both reports, the compound presented potent cytotoxicity towards mouse neuro-2a neuroblastoma cells (LD_50_ = 1.2 µM) [30] and human NCI-H460 lung cancer cells (IC_50_ = 153 nM) [31], which is in line with the strong reduction of cell viability observed in our assays (Table 1). Leptochelin was isolated from the Red Sea *Leptolyngbya* sp. RS02 and from the Indonesian *Leptolyngbya* sp. HB_3/1/2, which share identical 16S rRNA gene sequences even though they were collected in different geographical locations. Interestingly, our strain, unidentified Synechococcales LEGE 181150, was collected from a marine environment in the Cape Verde archipelago and falls in a subclade apart from the *Leptolyngbya* strains (Figure 1), suggesting the compound to be produced by a different genus of cyanobacteria. Nevertheless, all these locations fall in the tropical region, which might suggest an ecological role subjacent to the production of this compound.

For fraction C of *Phormidium* sp. LEGE 15488, there were no mass features with striking values of fold change. This fact could be explained by the similarity in composition to fraction LEGE 15488_D that was included in group C (Figure S3). Thus, for the fraction LEGE 15488_C, the ions with the highest fold change will most probably be the ones responsible for the cytotoxic activity. As such, three protonated molecules were cherry-picked (Table 2). For these molecules, we could not retrieve any dereplication results either using the GNPS tools or manual search in the databases (Dictionary of Natural Products and CyanoMetDB). For the protonated molecule at *m*/*z* 1520.7861, the ClassyFire categories Direct Parent (descriptor for the largest structural feature that defines a compound) and Molecular Framework (descriptor for overall aliphaticity/aromaticity and number of cycles) suggested this compound to have a scaffold of the cyclic peptide-type containing aromatic amino acids. In addition to this in silico prediction, the presence of the doubly charged ion at *m*/*z* 760.8961 [M+2H]^2+^ also reinforces the possible large structure of this compound. Considering these observations and given the taxonomic position of *Phormidium* sp. LEGE 15488 and *Phormidium* sp. LEGE 05292 (Figure 1), we hypothesize that this mass could correspond to an undescribed portoamide-type compound with a proposed molecular formula of C_73_H_109_N_13_O_22_ (calculated for 1519.7810). As for the parent mass 858.5795 [M+H]^+^, it was found to be associated with the ESI in-source fragments at *m*/*z* 331.2010 and 528.3863. For the latter, the in silico annotation can give insights into the nature of this molecule, as it was categorized as a possible cyclic depsipeptide without aromatic amino acids (Table 2).

In this group of cytotoxic fractions, it is worth noting the following aspects: the group is mainly constituted by polar fractions (fraction C); the in silico chemical classification predicted the significant mass features to have a peptide-type scaffold (Figure 4B, Table 2); and the in silico dereplication lead to the putative annotation/identification of known peptides whose cytotoxic activity towards cancer cells had been previously described. Given that these predictions worked correctly with *Phormidium* sp. LEGE 05292 (strain producer of portoamides), we hypothesize that the strains *Phormidium* sp. LEGE 15488 (Amazon River, Brazil; Table S1) and the unidentified Nostocales LEGE 17548 (Mira lagoon, Beira Litoral, Portugal; Table S1) might be potential producers of cytotoxic peptide-type compounds. Furthermore, the strains *Gloeothece* sp. LEGE 16572 (isolated from a fountain, Monchique, Portugal; Table S1) and unidentified Synechococcales LEGE 181150 have potential for the discovery of totally unknown structures.

### 2.4. Group B: Metabolomics Analysis and Dereplication of the Putative Active Molecules

**Figure 6:**
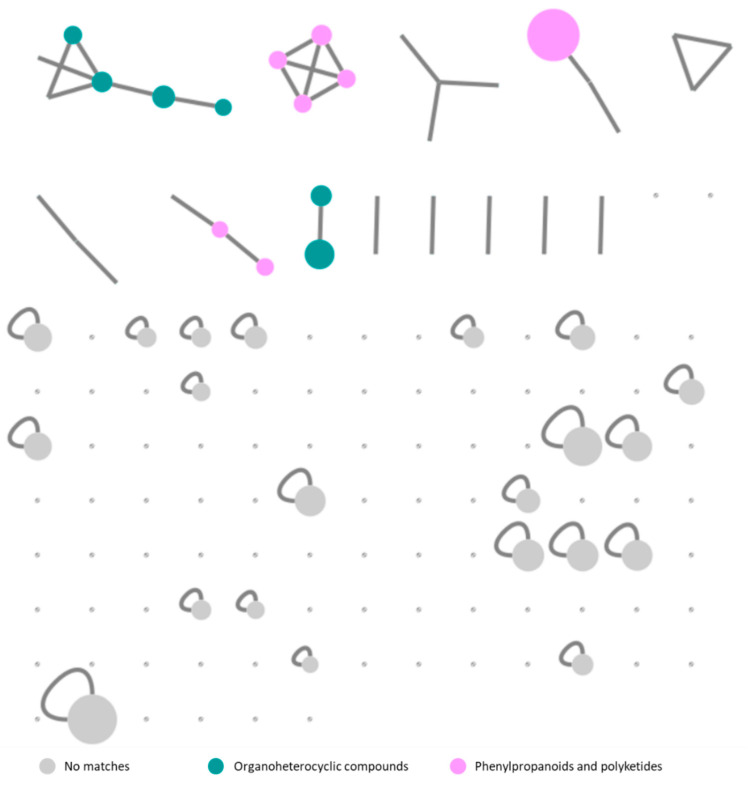
Feature-based molecular network of groups B/C annotated with MolNetEnhancer workflow. The nodes are color-coded accordingly to the ClassyFire super class classification, and their size is related to their fold change log2(FC) value.

The same untargeted metabolomics approach described above was applied for group B. The fold change analysis highlighted 34 mass features with log2(FC) between 2.14 and 17.13. Fifteen nodes were characterized, according to ClassyFire superclass, as organoheterocyclic compounds all belonging to the tetrapyrroles and derivatives class (23%) and phenylpropanoids and polyketides (21%) (Figure 6). Contrary to group A, in group B there were no mass features with high fold change values. In fact, the only three mass features that presented log2(FC) higher than 10 were 636.4814, 1245.5650, and 1267.5473. The *m*/*z* 636.4814 (12.9 min; log2(FC) = 17.14) was found in samples LEGE 16502_E and LEGE 15546_D, being characterized as a potential macrolide-type compound. However, its manual query on the mass databases did not retrieve any identification. 

The mass features 1245.5650 and 1267.5473, respectively, with log2(FC) of 15.85 and 11.59, were found to be the [2M+H]^+^ and [2M+Na]^+^ ions of the protonated molecule at *m*/*z* 623.2865 (11.68 min; log2(FC) = 7.54), predicted as a tetrapyrrole-type molecule. Despite this classification, the putative annotation via GNPS tools was not successful. Thus, 13^2^-hydroxy-phaeophorbide a methyl ester, was tentatively identified by manual search in the Dictionary of Natural Products and study of its MS2 fragmentation pattern (Table 3, Figure S4). This compound was found to be one of the main components of the samples LEGE 16572_D, LEGE 15546_D, and LEGE xx358_D (Figure 6, Table 3). This putative pheophorbide appeared clustered with a protonated molecule at *m*/*z* 609.2706 (11.30 min; log2(FC) = 3.90). The difference of 14.01 Da between the molecules suggested the loss of a methyl group, and thus was tentatively identified as 13^2^-hydroxy-pheophorbide a. This molecule was found in the environmental sample of a cyanobacterial mat (JM1_amb_E) and in the strain *Brasilonema* sp. LEGE 16502 (LEGE 16502_E). Moreover, further manual dereplication led to the tentative identification of other pheophytins and pheophorbides (Table 3). The lack of GNPS annotation for these compounds might be due to the fact that the masses deposited in the GNPS database were acquired in low resolution mass spectrometers, and thus, did not match with our search criteria. 

These pheophytins and pheophorbides are products of the degradation pathway of chlorophyll a, and their anticancer activity has been widely reported [32] Such molecules are commonly found in photosynthetic organisms, which suggests that the bioactivity results obtained for group B could be related to a higher content of these compounds in the fractions. Future studies will help to elucidate this observation and address possible ecological relationships.

## 3. Materials and Methods

### 3.1. Cyanobacteria Culture Conditions

The 60 cyanobacterial strains were obtained from the Blue Biotechnology and Ecotoxicology Culture Collection (LEGE-CC) (Table S1). To establish the natural products library, these microorganisms were cultured up to 4 L, in the appropriate growth media, and maintained under standard laboratory conditions: 25 °C with light/dark cycle of 14/10 h at a light intensity of 10–30 μmol photons m^−2^ s^−1^. The freshwater strains were cultured using Z8 medium, while the marine strains were grown using Z8 medium supplemented with 25‰ of synthetic sea salts (Tropic marin, Berlin, Germany) and 1‰ of vitamin B_12_ (Table S1). Depending on the strain, after 30 to 160 days of growth, the biomass was harvested either by centrifugation for unicellular strains, or by filtration for filamentous strains, through an appropriately sized mesh. All biomasses were freeze-dried (LyoQuest, Telstar, Terrassa, Spain) before organic extraction.

### 3.2. DNA Extraction, Amplification (PCR) and Sequencing

Twelve strains of cyanobacteria were characterized for the first time in this work (Figure 1). For taxonomic studies, these strains were grown in 50 mL culture flasks and cells were harvested after 15–20 days of cultivation. Genomic DNA was extracted using the Genomic DNA Mini Kit (Invitrogen, Waltham, MA, USA), according to the manufacturer’s instructions for Gram-negative bacteria. To obtain the complete sequence of 16S rRNA gene, PCR amplification was performed using the oligonucleotide primers set 27F [33] and 23S30R [34]. PCR reactions were performed in a final volume of 20 μL containing 1× Green GoTaq Flexi Buffer, 2.5 mM of MgCl_2_, 125.0 mM of each deoxynucleotide triphosphate, 1.0 μM of each primer, 0.5 U of GoTaq Flexi DNA Polymerase (Promega, Madison, WI, USA), 10 mg mL^−1^ of bovine serum albumin (BSA), and 10–30 ng of template DNA, on a TProfessional Standard thermal cycler (Biometra, Göttingen, Germany). The PCR conditions were as follows: initial denaturation at 94 °C for 5 min, followed by 10 cycles of denaturation at 94 °C for 45 s, annealing at 57 °C for 45 s, and extension at 72 °C for 2 min, followed by 25 cycles of denaturation at 92 °C for 45 s, annealing at 54 °C for 45 s, and extension at 72 °C for 2 min with a final elongation step at 72 °C for 7 min. The PCR reactions were performed in duplicate. PCR products were separated by 1.5% agarose gel stained with SYBR^®^ safe (Invitrogen, Waltham, MA, USA) and DNA fragments with the expected size were excised and purified using NZYGelpure (NzyTech, Genes and Enzymes, Lisbon, Portugal) according to the manufacturer′s instructions. Since the sequences were obtained by direct sequencing of purified amplicons, internal primers CYA359F, CYA781R [35], and 1494R [33] were used to improve the quality of the sequences. The sequencing was performed at GATC Biotech (Ebersberg, Germany) and the nucleotide sequences obtained were manually inspected for quality and assembled using the Geneious 11.1.5 software (Biomatters Ltd., Auckland, New Zealand). Possible chimera formation during the sequences was checked using the software DECIPHER [36] before any phylogenetic analysis. Sequences obtained were inserted in the BLASTn (Basic Local Alignment and Search Tool for Nucleotides) database and the results were analyzed. The sequences associated with this study were deposited in the GenBank database under the accession numbers MW790910 to MW790921 (Table S1).

### 3.3. Phylogenetic Analysis

A total of 146 sequences were used in the final analysis, including 2 strains of *Gloeobacter violaceus* as outgroup, 85 sequences of cyanobacteria including type and reference strains retrieved from GenBank (National Center for Biotechnology Information, NCBI, Bethesda, MD, USA), and 59 sequences of LEGE-CC strains from which 12 were obtained in this work. Multiple sequence alignment was constructed using ClustalW in MEGA7 [37,38], and sequences were manually proofread and edited. Maximum likelihood (ML) analysis was carried out using substitution model GTR+G+I according to the Bayesian information criterion (BIC) and Akaike information criterion (AIC) scores with 1000 bootstrap resampling replicates using the MEGA7 software [38]. The final phylogenetic tree was edited on iTOL (Interactive Tree of Life) [39].

### 3.4. Cyanobacterial Natural Products Library

The LEGE-NPL (natural products library) solid inventory is composed of crude extracts. Thus, freeze-dried biomass was extracted three times with MeOH, with a sonication step of 5 min in between extractions, and was filtered and concentrated at 30 °C, using a rotary evaporator. The yields of extraction are described in the Supplementary Material (Figure S1). The extracts were then fractionated by reverse-phase HPLC in a Waters Alliance e2695 Separations Module instrument, coupled to a photodiode array detector (Waters 2998 PDA) and an automatic Waters Fraction Collector III (Waters, Mildford, MA, USA). Each crude was injected at 40 mg mL^−1^ (500 µL; 1 mL loop) and separated on an ACE 10 C8 column (50 ×10 mm, ACE, Reading, UK), using a H_2_O:MeCN gradient (Table 4). Hence, each cyanobacterial extract was chromatographed into eight fractions (4 mL final volume, named A–H) into 48-deep well plates (Riplate, Ritter, Schwabmünchen, Germany), which were then dried on a CentriVap Concentrator (LabConco, Kansas City, MO, USA). These fractions were solubilized in 500 µL of DMSO and transferred to 96-deep well microplates (Nest Scientific, Woodbridge Township, NJ, USA) and stored at −80 °C, thus forming the LEGE-NPL liquid library (mother plates).

### 3.5. Cell Culture

The human colon carcinoma cell line HCT 116 was obtained from Sigma-Aldrich (St. Louis, MO, USA) and the human brain endothelial cell line hCMEC/D3 was kindly donated by Dr. P. O. Courad (INSERM, Paris, France). HCT 116 was cultured with McCoy′s 5A medium (CarlRoth, Kasruw, Germany) and hCMEC/D3 with Dulbecco’s modified Eagle medium (DMEM) (Gibco, Thermo Fisher Scientific, Waltham, MA, USA), both supplemented with 10% of fetal bovine serum (Biochrom, Berlin, Germany), 1% of penicillin/streptomycin (Biochrom, Berlin, Germany), and 0.1% of amphotericin (GE Healthcare, Little Chafont, Buckinghamshire, UK). Both cell lines were grown at 37 °C with 5% CO_2_ atmosphere.

### 3.6. Bioactivity Screening Using 2D Cell Models

The HCT 116 and hCMEC/D3 cells were seeded on 96-well plates, at a density of 3.3 × 10^4^ cells mL^−1^ for 24 h. Then, the cells were incubated with 25 µg mL^−1^ of LEGE-NPL fractions (0.5% DMSO final concentration) and 1.25 µM of staurosporine (positive control) for 48 h. After this period of exposure, cell viability was evaluated by the MTT colorimetric assay (3-(4,5-dimethylthiazol-2-yl)-2,5-diphenyltetrazolium bromide). Thus, the cells were incubated with 20 µL of MTT reagent, at a final concentration of 200 µg mL^−1^ over 3–4 h, and afterwards 100 µL of DMSO was used to dissolve formazan crystals. Absorbance was read at 550 nm on a multi-detection microplate reader (Synergy HT, Biotek, Bart Frederick Shahr, Germany). All assays were repeated three times. Cell viability was calculated using the following formula:

$$\%\;cell\;viability\;\left(to\;negative\;control\right)=\frac{\;\bar{x}\;(Absorbance_{sample})}{\;\bar{x}\;(Absorbance_{negative\;control})}\times 100$$

### 3.7. Bioactivity Screening Using 3D Cell Models

The cancer spheroids were produced using the scaffold-free liquid-overlay technique [40]. Briefly, 200 µL of McCoy′s medium with a HCT 116 cell density of 5 × 10^4^ cells mL^−1^ was added to ultra-low attachment round-bottom 96-well plates (Costar, Corning, New York, NY, USA). Cells were allowed to settle for 30 min, at room temperature, and then incubated for 5 days, at 37 °C under 5% CO_2_ atmosphere, until the spheroids were properly formed. After renewal of the culture medium, the spheroids were incubated with 25 µg mL^−1^ of LEGE-NPL fractions (0.5% DMSO final concentration) and 1.25 µM staurosporine (positive control) for 96 h. Cell viability was evaluated using the acid phosphatase assay. Hence, media was removed, the wells were carefully washed with PBS, and the spheroids were incubated for 2 h in 100 µL of p-nitrophenyl phosphate (2 mg mL^−1^) in sodium acetate buffer (0.1 M). To stop the reaction, 10 µL of NaOH (1 N) was added to each well and the absorbance was read at 405 nm on a multi-detection microplate reader (Synergy HT, Biotek, Bart Frederick Shahr, Germany). All assays were performed in triplicate and cell viability was calculated according to the formula above. Graphics were designed using Plotly Chart Studio [41].

### 3.8. Untargeted Metabolomics Analysis

To identify the putative cytotoxic compounds, an untargeted metabolomics approach was performed. Groups A and B were constituted by the active fractions of the study (Table 1, Figure 4). Group C was constituted by 12 fractions that were not considered active: JM5_amb_D, JM5_amb_E, LEGE 06078_D, LEGE 07092_D, LEGE 07167_C, LEGE 07167_D, LEGE 07167_E, LEGE 08333_D, LEGE 15488_D, LEGE 181148_E, LEGE 181148_F, and LEGE 181149_D. The liquid chromatography-high resolution electrospray ionization tandem mass spectrometry (LC-HRESIMS/MS) data were acquired on a system composed of a Dionex UltiMate 3000 HPLC with a MWD-3000RS UV/VIS detector, coupled to a Q Exactive Focus mass spectrometer controlled by Xcalibur 4.1 software (Thermo Fisher Scientific, Waltham, MA, USA). Then, 5 µL (1 mg mL^−1^ in MeOH) was separated on an ACE UltraCore 2.5 SuperC18 column (75 × 2.1 mm, ACE, Reading, UK), at 40 °C, using a gradient from 99.5 to 10% H_2_O/MeOH/formic acid (95:5:0.1, *v*/*v*) to 0.5 to 90% isopropanol/MeOH/formic acid (95:5:0.1, *v*/*v*) for 9.5 min, maintaining the last mixture until 15.5 min before returning to the initial conditions, with a flow rate of 0.35 mL min^−1^ [42]. The UV absorbance was monitored at 254 nm. HRESIMS-MS was obtained in positive mode using a capillary temperature of 262.5 °C, spray voltage of 3.5 kV, full MS scan at the resolution of 70,000 FWHM (*m*/*z* range of 150–2000), and data dependent MS^2^ (ddMS^2^, Discovery mode) at the resolution of 17,500 FWHM (isolation window used was 3.0 amu and normalized collision energy was 35). Raw data files were converted to the mzML format with MSConvert, using the parameters recommended for the Global Natural Product Social Molecular Networking (GNPS) [43]. MZmine 2 v.2.53 (http://mzmine.github.io/) was used to generate the quantification file used in the fold change analysis of MetaboAnalyst 5.0 (https://www.metaboanalyst.ca/), and to generate the MS^2^ spectral summary file and quantification file for feature-based molecular networking (parameters used in MZmine 2 for mass feature detection, chromatogram building, and alignment can be found in Table S2). The appropriate files were uploaded to the GNPS web platform, and the feature-based molecular networking (FBMN) was constructed using the default settings. This molecular network was analyzed with the integrated GNPS tools DEREPLICATOR [22], MS2LDA [23], and Network Annotation Propagation (NAP) [24], which were all combined via the MolNetEnhancer [25] workflow. The web links that gave origin to the results are provided in Table S3 and the structure database used for NAP can be found as Supplementary Material. For the fold change analysis with MetaboAnalyst 5.0, the data was uploaded in comma separated values (.csv) format, with 18 unpaired samples (fractions) in columns and mass features in rows (474 mass features for group A/C and 137 mass features for group B/C; PCA and fold change charts are shown in Figure S5). No data filtering or data normalization was performed, and missing values were replaced by 1. Cytoscape 3.8.2 was used to combine the GNPS and MetaboAnalyst results and visualize the resulting molecular network. Manual dereplication was done by using the Dictionary of Natural Products 30.1 Chemical Search (https://dnp.chemnetbase.com) and CyanoMetDB [29].

## 4. Conclusions

Cyanobacteria have acquired an indisputable role in natural products drug discovery. Our in-house culture collection of cyanobacteria (LEGE-CC) harbors a great potential to explore for biotechnological applications, but in prior works, this was often very laborious and unsuccessful. Therefore, there was a need to develop a new strategy to access the chemical richness of LEGE-CC in a more expedited way. In summary, the semiautomated HPLC fractionation of 64 crudes generated 512 fractions that were tested for their cytotoxic potential using different cell models. The conjugation of monolayer assays and 3D cancer spheroids lead to the selection of 11 active fractions, whose chemical space was studied using an untargeted metabolomics approach. The putative annotation and identification of several cytotoxic compounds contributed to expanding the knowledge of the biochemical composition of 7 LEGE-CC strains that were characterized herein for the first time. This study was relevant to prioritize the strains with potential to discover compounds of unknown structure, work that will be addressed in the near future.

## Figures and Tables

**Table 1:** Cytotoxic profile of the selected fractions of group A, B, and C.

	Fractions	Cell Viability (%)		
		3D HCT 116	2D HCT 116	2D hCMEC/D3
Group A	LEGE 16572_C	57.54 ± 20.74	21.30 ± 8.79	12.97 ± 5.14
	LEGE 17548_C	58.05 ± 11.10	16.33 ± 8.50	16.67 ± 11.84
	LEGE 17548_D	58.74 ± 13.40	27.08 ± 17.23	36.52 ± 16.23
	LEGE 15488_C	65.16 ± 10.30	25.69 ± 21.02	14.51 ± 8.65
	LEGE 181150_D	75.95 ± 4.25	16.40 ± 3.26	13.80 ± 3.14
	LEGE 05292_C	68.32 ± 4.60	9.13 ± 2.64	12.83 ± 3.35
Group B	LEGE XX358_D	72.28 ± 16.86	50.11 ± 20.43	44.60 ± 14.66
	LEGE 16572_D	72.34 ± 6.05	58.15 ± 23.40	56.18 ± 19.94
	JM1 Amb_D	75.25 ± 7.14	55.30 ± 3.11	22.77 ± 5.59
	LEGE 15546_D	78.51 ± 7.16	58.44 ± 18.34	59.17 ± 15.65
	LEGE 16502_E	81.53 ± 13.30	71.38 ± 13.24	58.29 ± 7.41
	JM1 Amb_E	82.07 ± 3.02	56.16 ± 12.80	21.15 ± 5.76
Group C	JM5_amb_D	90.07 ± 1.24	52.49 ± 5.34	31.85 ± 4.39
	JM5_amb_E	94.92 ± 5.66	48.11 ± 12.12	55.44 ± 7.66
	LEGE 06078_D	99.29 ± 7.89	46.51 ± 12.42	69.21 ± 15.63
	LEGE 07092_D	94.96 ± 6.94	47.77 ± 3.62	55.86 ± 3.63
	LEGE 07167_C	85.76 ± 2.54	25.86 ± 1.36	56.83 ± 4.00
	LEGE 07167_D	82.74 ± 3.25	49.51 ± 1.90	10.93 ± 2.87
	LEGE 07167_E	99.70 ± 4.71	47.34 ± 1.59	53.96 ± 3.48
	LEGE 08333_D	106.90 ± 5.38	85.02 ± 1.57	80.56 ± 5.12
	LEGE 15488_D	99.09 ± 4.96	36.76 ± 23.26	20.17 ± 15.65
	LEGE 181148_E	93.04 ± 6.94	64.14 ± 14.46	48.58 ± 4.75
	LEGE 181148_F	93.49 ± 6.54	71.52 ± 20.38	48.44 ± 2.02
	LEGE 181149_D	94.01 ± 7.29	64.47 ± 22.29	40.25 ± 2.66
Selection threshold	LEGE 05292_C + 3*σ*	82.12	17.03	
Staurosporine		31.67 ± 6.84	20.07 ± 4.40	12.05 ± 2.55

**Table 2:** Characterization and annotation of the putative cytotoxic molecules present in the active fractions of group A.

*m*/*z*	Isotope/ Fragments	Rt (min)	Log2(FC)	Super Class	Direct Parent	Molecular Framework	Putative Annotation
LEGE 05292_C							
1313.6991 [M+H]^+^		7.21	25.71	3	8	9	Portoamide C C_62_H_96_N_12_O_19_ Δ 0.27 ppm
1532.7887 [M+H]^+^		7.51	28.18	2	10	9	Portoamide A C_74_H_109_N_13_O_22_ Δ 0.27 ppm
1502.7780 [M+H]^+^		7.74	26.34	2	10	9	Portoamide B C_73_H_107_N_13_O_21_ Δ 0.18 ppm
LEGE 17548_C							
1154.6172 [M+Na]^+^		8.73	26.07	-	-	-	[Minutissamide A + CH_3_] * C_52_H_85_N_13_O_15_ Δ −1.55 ppm
1118.6187 [M+H]^+^		8.76	26.86	3	8	11	Minutissamide A C_51_H_83_N_13_O_15_ Δ −1.55 ppm
LEGE 17548_C, LEGE 17548_D and LEGE 16572_C							
762.5468 [M+H]^+^		12.60	27.91	-	-	-	-
LEGE 16572_C							
703.5092 [M+H]^+^		12.31	27.33	-	-	-	-
LEGE 181150_D							
895.0778	897.0759 [M+2 isotope]	7.92	25.98	-	-	-	Leptochelin
851.1284	853.1257 [M+2 isotope]	7.81	17.43	-	-	-	Leptochelin-like *
1011.8493		14.57	27.92	-	-	-	-
LEGE 15488_C							
655.3808 [M+H]^+^		5.73	8.27	-	-	-	-
858.5795 [M+H]^+^		7.29	8.10	-	-	-	-
	331.2010	7.28	8.15	-	-	-	-
	528.3863	7.28	9.05	3	12	11	-
1520.7861 [M+H]^+^		7.87	5.07	2	10	9	Portoamide-like * C_73_H_109_N_13_O_22_ Δ −1.44 ppm

2—Organic polymers; 3—organic acids and derivatives; 8—cyclic peptides; 9—aromatic heteropolycyclic compounds; 10—polypeptides 11—aliphatic heteropolycyclic compounds; 12—Cyclic depsipeptides; * tentative identification.

**Table 3:** Characterization and annotation of the putative cytotoxic molecules present in the active fractions of group B.

*m*/*z*	Rt (min)	Log2(FC)	Super Class	Precursor Intensity	Tentative Identification
LEGE 16572_D, LEGE 15546_D and LEGE xx358_D					
653.2971 [M+H]^+^	12.12	7.72	1	1.19 × 10^10^	15^1^-hydroxy-lactone-pheophorbide a ethyl ester C_37_H_40_N_4_O_7_ Δ 0.19 ppm
623.2865 [M+H]^+^	11.68	7.54	1	2.55 × 10^10^	13^2^-hydroxy-phaeophorbide a methyl ester C_36_H_38_N_4_O_6_ Δ 0.14 ppm
639.2813 [M+H]^+^	11.88	5.99	2	2.44 × 10^9^	15^1^-hydroxy-lactone-pheophorbide a methyl ester C_36_H_38_N_4_O_7_Δ −0.04 ppm
LEGE 16502_E, JM1_amb_E					
593.2759 [M+H]^+^	11.77	6.99	2	2.97 × 10^10^	pheophorbide a C_35_H_36_N_4_O_5_ Δ 0.09 ppm
535.2704 [M+H]^+^	12.24	4.18	2	3.85 × 10^9^	pyrophaeophorbide a C_33_H_34_N_4_O_3_Δ 0.06 ppm
609.2706 [M+H]^+^	11.29	3.90	1	5.22 × 10^8^	13^2^-hydroxy-phaeophorbide a C_35_H_36_N_4_O_6_ Δ −0.26 ppm
All samples					
903.5618 [M+H]^+^	14.41	4.58	2	1.47 × 10^10^	15^1^-hydroxy-lactone-phaeophytin a C_55_H_74_N_4_O_7_Δ −1.36 ppm
887.5664 [M+H]^+^	14.30	3.67		4.80 × 10^10^	13^2^-hydroxy-pheophytin a C_55_H_74_N_4_O_6_Δ −1.93 ppm
JM1_amb_D, JM1_amb_E, LEGE 16502_E					
917.5777 [M+H]^+^	14.70	2.16	2	2.02 × 10^9^	13-methyldioxy-phaeophytin a/ ficusmicrochlorin B C56H76N4O7 Δ −1.07 ppm

1—No Matches, 2—Organic polymers.

**Table 4:** HPLC chromatographic and collection program for generating the fractions for LEGE-NPL liquid inventory.

Time (min)	Flow (mL·min^−1^)	MeCN (%)	H_2_O (%)	Collection Time (min)	Fraction
0.0	3.0	10	90	1.00–2.30	A
2.0	3.0	80	20	2.30–3.60	B
3.0	3.0	80	20	3.60–4.90	C
4.0	3.0	100	0	4.90–6.20	D
8.9	3.0	100	0	6.20–7.50	E
9.2	3.5	100	0	7.50–8.80	F
12.0	3.5	100	0	8.80–10.36	G
12.3	3.0	100	0	10.36–11.50	H
14.0	3.0	100	0		
15.0	3.0	10	90		
18.0	3.0	10	90		

## Data Availability

The data presented in this study are available in the Supplementary Materials.

## Supplementary Materials

The following are available online at https://www.mdpi.com/article/10.3390/md19110633/s1, Table S1: List of LEGE-CC strains and environmental samples used in this work. Asterisks (*) represent sequences that were obtained for this work. Table S2: Parameters used in MZmine 2 for mass feature detection, chromatogram building and feature alignment for comparison of group A with C and group B with C. Table S3: GNPS jobs used for the construction of the molecular networks. Figure S1: Boxplot representing the max, min, median and mean amounts of lyophilized biomass (A), MeOH extract (B) and final yield (C). Figure S2: IC50 graphs from the optimization of the MTT and acid phosphatase assays on 3D spheroids of the HCT 116 human colon carcinoma cell line. A range of concentrations, from 0.1 nM to 10 µM, of the anticancer drug staurosporine was used to determine the most sensitive method for evaluating cytotoxicity in cell spheroids. Figure S3: Total ion chromatograms of fractions LEGE 15488_C (upper) and LEGE 15488_D (lower). Figure S4: MS/MS spectrum of the protonated molecule at m/z 623.2865. The MS2 fragments and molecular formula were consistent with the tentative identification of 13^2^-hydroxy-phaeophorbide a methyl ester. Figure S5: Principal component analysis (PCA) and fold change plots of the untargeted metabolic analysis of group A/C (A) and group B/C (B).
