# Supplementary material for: Uncovering the Bioactive Potential of a Cyanobacterial Natural Products Library Aided by Untargeted Metabolomics
Source: Mar Drugs. 2021 Nov 12;19(11):633. doi: 10.3390/md19110633 (PMC8624515; doi:10.3390/md19110633)
Supplement: Supplementary file 1 [file marinedrugs-19-00633-s001.zip › Supplementary information_Ferreira et al.pdf]

Supplementary information

Uncovering the Bioactive Potential of a Cyanobacterial Natural Products Library Aided by Untargeted Metabolomics

Leonor Ferreira[1], João Morais[1,2], Marco Preto[1], Raquel Silva[1], Ralph Urbatzka[1], Vitor Vasconcelos[1,2], Mariana Reis[1,*]

[1] Interdisciplinary Centre of Marine and Environmental Research (CIIMAR/CIMAR), Terminal de Cruzeiros do Porto de Leixões, University of Porto, 4450-208 Matosinhos, Portugal

[2] Faculdade de Ciências, Universidade do Porto, Rua do Campo Alegre, Edifício FC4, 4169-007 Porto, Portugal

* Correspondence: mreis@ciimar.up.pt

Table of contents

Table S1. List of LEGE-CC strains and environmental samples used in this work. Asterisks (*) represent sequences that were obtained for this work. S2

Table S2. Parameters used in MZmine 2 for mass feature detection, chromatogram building and feature alignment for comparison of group A with C and group B with C. S5

Table S3. GNPS jobs used for the construction of the molecular networks S6

Figure S1: Boxplot representing the max, min, median and mean amounts of lyophilized biomass(A), MeOH extract (B) and final yield (C) S7

Figure S2: IC50 graphs from the optimization of the MTT and acid phosphatase assays on 3D spheroids of the HCT 116 human colon carcinoma cell line. A range of concentrations, from 0.1 nM to 10 µM, of the anticancer drug staurosporine was used to determine the most sensitive method for evaluating cytotoxicity in cell spheroids. S8

Figure S3. Total ion chromatograms of fractions LEGE 15488_C (upper) and LEGE 15488_D (lower). S8

Figure S4. MS/MS spectrum of the protonated molecule at *m/z* 623.2865. The MS2 fragments and molecular formula were consistent with the tentative identification as 13^2-hydroxy-phaeophorbide a methyl ester. S9

Figure S5. Principal Component Analysis (PCA) and Fold Change plots of the untargeted metabolic analysis of group A/C (A) and group B/C (B). S10

Table S1. List of LEGE-CC strains and environmental samples used in this work. Asterisks (*) represent sequences that were obtained for this work.

Strain Identification	Order	Accession Number	Environment	Growth media	Habitat|Sample Description	Location	Country
Brasilonema sp. LEGE 16502*	Nostocales	MW790911	freshwater	Z8	floodgate wall, scraping	Rio Douro	Portugal
cf. *Oculatella* sp. LEGE 06141	Synechococcales	KU951789	marine	Z8 + 25‰ Salt TM+ 1‰B12	intertidal zone, on green macroalga	Praia da Luz, Lagos	Portugal
cf. *Oxynema acuminatum* LEGE 06072	Oscillatoriales	HM217077	brackish	Z8	mesotidal zone, benthic	Vouga estuary, Ria de Aveiro, São Jacinto	Portugal
cf. *Oxynema acuminatum* LEGE 06078	Oscillatoriales	HM217075	brackish	Z8	mesotidal zone, benthic	Douro estuary, Vila Nova de Gaia	Portugal
cf. *Phormidesmis* sp. LEGE 10370	Synechococcales	JQ927344	marine	Z8 + 25‰ Salt TM+ 1‰B12	intertidal zone, on a marine sponge	Praia da Memória	Portugal
cf. *Romeria* sp. LEGE 06013	Synechococcales	KU951673	marine	Z8 + 25‰ Salt TM+ 1‰B12	intertidal zone, wave-exposed rock	Praia da Foz do Arelho, Caldas da Rainha	Portugal
Chroococcopsis sp. LEGE 07187	Pleurocapsales	HQ832904	marine	Z8 + 25‰ Salt TM+ 1‰B12	intertidal zone, on a *Gibbul*a sp. shell	Praia de Moledo, Caminha	Portugal
Coleofasciculus sp. LEGE 07092	Oscillatoriales	HM217070	brackish	Z8	mesotidal zone, benthic	Vouga estuary, Ria de Aveiro, São Jacinto	Portugal
Cyanobium sp. LEGE 06015	Synechococcales	KU951688	marine	Z8 + 25‰ Salt TM+ 1‰B12	intertidal zone, on a *Patella* sp. shell	Baleal Norte, Ferrel	Portugal
Cyanobium sp. LEGE 06097	Synechococcales	HQ832928	marine	Z8 + 25‰ Salt TM+ 1‰B12	intertidal zone, on a green macroalga	Praia do Martinhal, Vila do Bispo	Portugal
Cyanobium sp. LEGE 06098	Synechococcales	KC469572	marine	Z8 + 25‰ Salt TM+ 1‰B12	intertidal zone, on a green macroalga	Praia do Martinhal, Vila do Bispo	Portugal
Cyanobium sp. LEGE 06139	Synechococcales	KC469574	marine	Z8 + 25‰ Salt TM+ 1‰B12	intertidal zone, on a *Mytilu*s sp. shell	Praia da Aguda, Arcozelo	Portugal
Cyanobium sp. LEGE 07183	Synechococcales	KU951702	marine	Z8 + 25‰ Salt TM+ 1‰B12	tide puddle, rock surface scraping	Praia de Olhos d'Água, Albufeira	Portugal
Dolichospermum sp. LEGE 00263	Nostocales	KU951721	freshwater	Z8	water sample	Maranhão dam reservoir, Benavila	Portugal
Geitlerinema sp. LEGE 11390	Oscillatoriales	KT730209	hypersaline	Z8 + 25‰ Salt TM+ 1‰B12	upper layer of a microbial mat	Araruama (main) lagoon, Rio de Janeiro	Brazil
Geitlerinema sp. LEGE 11391	Oscillatoriales	KT730210	hypersaline	Z8 + 25‰ Salt TM+ 1‰B12	upper layer of a microbial mat	Araruama (main) lagoon, Rio de Janeiro	Brazil
Geitlerinema sp. LEGE 11393	Oscillatoriales	KT730212	hypersaline	Z8 + 25‰ Salt TM+ 1‰B12	upper layer of a microbial mat	Pernambuco lagoon (Araruama system), Rio de Janeiro	Brazil
Geitlerinema sp. LEGE 11396	Oscillatoriales	KT730215	hypersaline	Z8 + 25‰ Salt TM+ 1‰B12	upper layer of a microbial mat	Araruama (main) lagoon, iRio de Janeiro	Brazil
Geitlerinema sp. LEGE 181148*	Oscillatoriales	MW790915	marine	Z8 + 25‰ Salt TM+ 1‰B12	rock surface scraping	Baía das Gatas, São Vicente Island	Cape Verde
Geminobacterium atlanticum LEGE 07459	Chroococcales	KU951882	marine	Z8 + 25‰ Salt TM+ 1‰B12	intertidal zone, on a *Patella* sp. shell	Praia do Martinhal, Vila do Bispo	Portugal
Gloeothece sp. LEGE 16572*	Chroococcales	MW790910	freshwater	Z8	fountain	Malhada Quente, Serra de Monchique	Portugal
Leptolyngbya ectocarpi LEGE 11425	Synechococcales	KT951671	marine	Z8 + 25‰ Salt TM+ 1‰B12	subtidal sample, epilithic (10-13m depth), less than 1 km off the shore	Pêlo Negro', diving spot near Leixões Harbour	Portugal
Leptolyngbya sp. LEGE 06361	Synechococcales	Not available	freshwater	Z8	biofilm, from a secondary decanter tank bank	wastewater treatment plant at Febros river, Vila Nova de Gaia	Portugal

Limnoraphis robusta LEGE XX358	Oscillatoriales	KU951735	freshwater	Z8	unknown	unknown	unknown
Lusitaniella coriacea LEGE 07167	Synechococcales	KU951853	marine	Z8 + 25‰ Salt TM+ 1‰B12	tide puddle, rock surface scraping	Praia de Lavadores, Canidelo	Portugal
Microcoleus sp. LEGE 16525*	Oscillatoriales	MW790913	freshwater	Z8	floodgate wall, scraping	Rio Douro	Portugal
Microcystis aeruginosa LEGE 91094	Chroococcales	KU950712	freshwater	Z8	pond, water sample	Lagoa de Mira	Portugal
Nodosilinea nodulosa LEGE 06102	Synechococcales	HQ832906	marine	Z8 + 25‰ Salt TM+ 1‰B12	tide pool, on a submerged stone	Praia de São Bartolomeu do Mar, Esposende	Portugal
Nodosilinea sp. LEGE 06009	Synechococcales	JF708121	marine	Z8 + 25‰ Salt TM+ 1‰B12	intertidal zone, epilithic	Praia da Foz do Arelho, Caldas da Rainha	Portugal
Nodosilinea sp. LEGE 06115	Synechococcales	KU951765	marine	Z8 + 25‰ Salt TM+ 1‰B12	intertidal zone, on a green macroalga	Praia da Luz, Lagos	Portugal
Nodosilinea sp. LEGE 07085	Synechococcales	HM217079	brackish	Z8	mesotidal zone, benthic	Douro estuary, Porto	Portugal
Nodosilinea sp. LEGE 181149*	Synechococcales	MW790919*	marine	Z8 + 25‰ Salt TM+ 1‰B12	extensive microbial mat on rocks	Salamansa, São Vicente Island	Cape Verde
Nostoc sp. LEGE 12449	Nostocales	KU951783	terrestrial	Z8	cycad coralloid root (host: *Encephalartos horridus*)	Botanical Garden of the University of Coimbra	Portugal
Nostoc sp. LEGE 12450	Nostocales	KU951784	terrestrial	Z8	cycad coralloid root (host: *Encephalartos horridus*)	Tropical Botanical Garden of Lisbon	Portugal
Nunduva sp. LEGE 07159	Nostocales	HQ832938	marine	Z8 + 25‰ Salt TM+ 1‰B12	tide pool, rock surface scraping	Praia de Burgau, Budens	Portugal
Pegethrix sp. LEGE 16528*	Synechococcales	MW790917	freshwater	Z8	convent channels	Arrábida	Portugal
Phormidium cf. *irriguum* LEGE 00055	Oscillatoriales	KU951790	freshwater	Z8	water sample	Imfout reservoir, Oum Rabiaa basin	Morocco
Phormidium sp. LEGE 00064	Oscillatoriales	Not available	freshwater	Z8	unknown	Lake Takerkoust	Morocco
Phormidium sp. LEGE 05292	Oscillatoriales	GU085101	freshwater	Z8	on an indoor aquarium wall	Porto	Portugal
Phormidium sp. LEGE 15488	Oscillatoriales	MF629805	freshwater	Z8	water sample, euphotic zone	Macapá, Amazon river	Brazil
Plectonema cf. *radiosum* LEGE 06114	Oscillatoriales	Not available	marine	Z8 + 25‰ Salt TM+ 1‰B12	intertidal zone, on a green macroalga	Praia da Luz, Lagos	Portugal
Pseudanabaena cf. *curta* LEGE 07169	Synechococcales	HQ832923	marine	Z8 + 25‰ Salt TM+ 1‰B12	intertidal zone, wave-exposed rock	Praia da Aguda, Arcozelo	Portugal
Sphaerospermopsis sp. LEGE 00249	Nostocales	KC989701	freshwater	Z8	water sample	Maranhão dam reservoir, campsite at Montargil	Portugal
Sphaerospermopsis sp. LEGE 02266	Nostocales	KU951801	freshwater	Z8	water sample	Maranhão dam reservoir, Benavila, Avis	Portugal
Sphaerospermopsis sp. LEGE 08334	Nostocales	KU951802	freshwater	Z8	water sample	Lake Zumpango	Mexico
Synechococcus nidulans LEGE 07171	Synechococcales	KU951805	marine	Z8 + 25‰ Salt TM+ 1‰B12	tide puddle, air-exposed rock surface scraping	Praia de Burgau, Budens	Portugal
Synechococcus sp. LEGE 07172	Synechococcales	HQ832950	marine	Z8 + 25‰ Salt TM+ 1‰B12	tide puddle, on a submerged stone	Praia de Olhos d'Água, Albufeira	Portugal
Synechocystis salina LEGE 00040	Chroococcales	KU951817	marine	Z8 + 25‰ Salt TM+ 1‰B12	intertidal zone, on a *Patella* sp. shell	Praia de Vila Praia de Âncora, Caminha	Portugal
Synechocystis sp. LEGE 06005	Chroococcales	KU951820	marine	Z8 + 25‰ Salt TM+ 1‰B12	sea water sample, coastal, surf zone	São Pedro de Moel	Portugal
Tildeniella sp. LEGE 16518*	Synechococcales	MW790918	freshwater	Z8	ditch	Exploratório - Centro Ciência Viva de Coimbra, Coimbra	Portugal

Toxifilum mysidocida LEGE 06108	Synechococcales	HQ832942	marine	Z8 + 25‰ Salt TM+ 1‰B12	tide puddle, rock surface scraping	Praia da Luz, Lagos	Portugal
Tychonema sp. LEGE 16526*	Oscillatoriales	MW790914	freshwater	Z8	water tank	Malhada Quente, Serra de Monchique	Portugal
unidentified Nostocales LEGE 17548*	Nostocales	MW790912	freshwater	Z8	lagoon	Lagoa de Mira, Mira	Portugal
unidentified Synechococcales LEGE 06070	Synechococcales	HM217074	brackish	Z8	mesotidal zone, benthic	Douro estuary, Porto	Portugal
unidentified Synechococcales LEGE 06144	Synechococcales	HQ832937	marine	Z8 + 25‰ Salt TM+ 1‰B12	intertidal zone, wave-sheltered zone, sand	Praia de Burgau, Budens	Portugal
unidentified Synechococcales LEGE 07163	Synechococcales	HQ832900	marine	Z8 + 25‰ Salt TM+ 1‰B12	intertidal zone, on a *Mytilus* sp. shell	Praia de Moledo, Caminha	Portugal
unidentified Synechococcales LEGE 08333	Synechococcales	KU951884	freshwater	Z8	water sample	Lake Zumpango	Mexico
unidentified Synechococcales LEGE 15546*	Synechococcales	MW790916	freshwater	Z8	water tank, effluent	Oliveira do Hospital	Portugal
unidentified Synechococcales LEGE 181150*	Synechococcales	MW790920	marine	Z8 + 25‰ Salt TM+ 1‰B12	rock surface scraping	Salamansa, São Vicente Island	Cape Verde
unidentified cyanobacterium LEGE 181151*	Chroococcales	MW790921	marine	Z8 + 25‰ Salt TM+ 1‰B12	Port ramp, scraping	Calhau, São Vicente Island	Cape Verde
JM1_amb (environmental sample)	n/a	n/a	marine	n/a	tide puddle, rock surface scraping	Baía das Gatas, São Vicente Island	Cape Verde
JM5_amb (environmental sample)	n/a	n/a	marine	n/a	tide puddle, rock surface scraping	Cova da Inglesa (Lazareto) , São Vicente Island	Cape Verde
VR2_amb (environmental sample)	n/a	n/a	marine	n/a	tide puddle, rock surface scraping	Baía das Gatas, São Vicente Island	Cape Verde
VV8_amb (environmental sample)	n/a	n/a	marine	n/a	tide puddle, rock surface scraping	Calhau, São Vicente Island	Cape Verde

Table S2. Parameters used in MZmine 2 for mass feature detection, chromatogram building and feature alignment for comparison of group A with C and group B with C.

**MZmine 2 workflow**	**Parameters used**	
1. Mass detection (mass detector centroid)	Noise level for MS1	1E5
	Noise level for MS2	1E3
2. ADAP chromatogram builder	Minimum group size in # of scans	5
	Group intensity threshold	3E5
	Minimum highest intensity	3E5
	m/z tolerance	5.0 ppm
3. Chromatogram deconvolution (local minimum search)	Chromatographic threshold	10%
	Search minimum in RT range	0.1
	Minimum relative high	10%
	Minimum absolute height	6E5
	Min ratio of peak top/edge	1
	Peak duration range (min)	0.05 – 3.00
	m/z range for MS2 scan pairing (Da)	0.01
	RT range for MS2 scan pairing (min)	0.2
4. Isotope grouping (Isotope peak grouping)	m/z tolerance	5.0 ppm
	Retention time	0.1
	Maximum charge	2
	Representative isotope	Most intense
5. Alignment (Join aligner)	m/z tolerance	5.0 ppm
	Weight for m/z	75
	Retention time tolerance	0.1 min
	Weight for RT	25
6. Filtering (Feature list row filter)	Keep only peaks with MS2 scan (GNPS)	
7. Gap filling (peak finder)	Intensity tolerance	10%
	m/z tolerance	5.0 ppm
	Retention time tolerance	0.1 min

Table S3. GNPS jobs used for the construction of the molecular networks

GNPS tool	Link of the job for Group A/C	Link of the job for Group B/C
Feature-based molecular network	https://gnps.ucsd.edu/ProteoSAFe/index.jsp?task=2a6808132a7647eda68e34d39e53f8c4	https://gnps.ucsd.edu/ProteoSAFe/status.jsp?task=63b0fd8424774a4e9e5afed20a659fac
DEREPLICATOR	https://gnps.ucsd.edu/ProteoSAFe/status.jsp?task=3359f38f4aa345e2abed42e73fd794af	https://gnps.ucsd.edu/ProteoSAFe/status.jsp?task=3e858ecd3bc9491d8096fe4022e487b0
MS2LDA	https://gnps.ucsd.edu/ProteoSAFe/index.jsp?task=41cf31d02d29420398be388c38fd51d3	https://gnps.ucsd.edu/ProteoSAFe/status.jsp?task=9f749fdaa8fb47ddbf5d57b2c0131594
Network Annotation Propagation (NAP)	https://gnps.ucsd.edu/ProteoSAFe/index.jsp?task=d143a06b06ab4f41bc40d3a1ffb2ccec	https://gnps.ucsd.edu/ProteoSAFe/status.jsp?task=f60c79693c3d4a55b8bbc5cea346d016
MolNetEnhancer	https://gnps.ucsd.edu/ProteoSAFe/index.jsp?task=7c66044d0db848798b448502df802ca2	https://gnps.ucsd.edu/ProteoSAFe/status.jsp?task=a4d1dd23b94248cca176689e4f1b7288

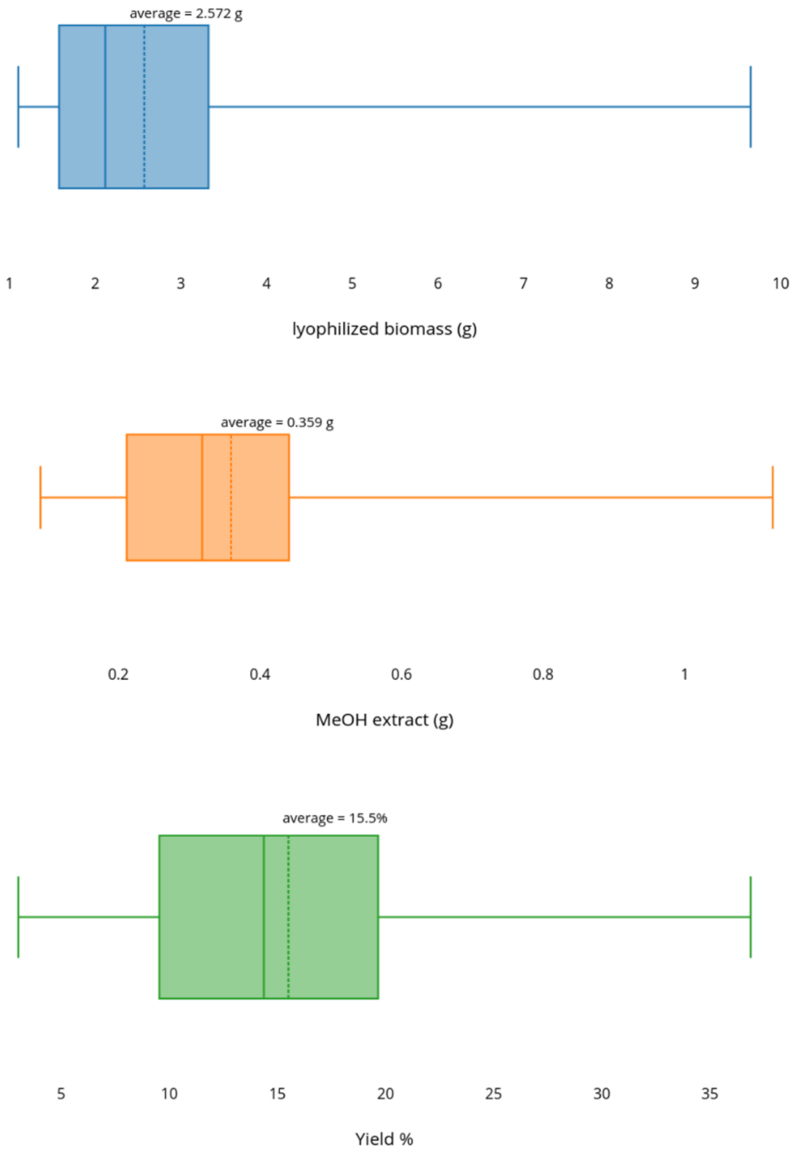

Figure S1: Boxplot representing the max, min, median and mean amounts of lyophilized biomass(A), MeOH extract (B) and final yield (C).

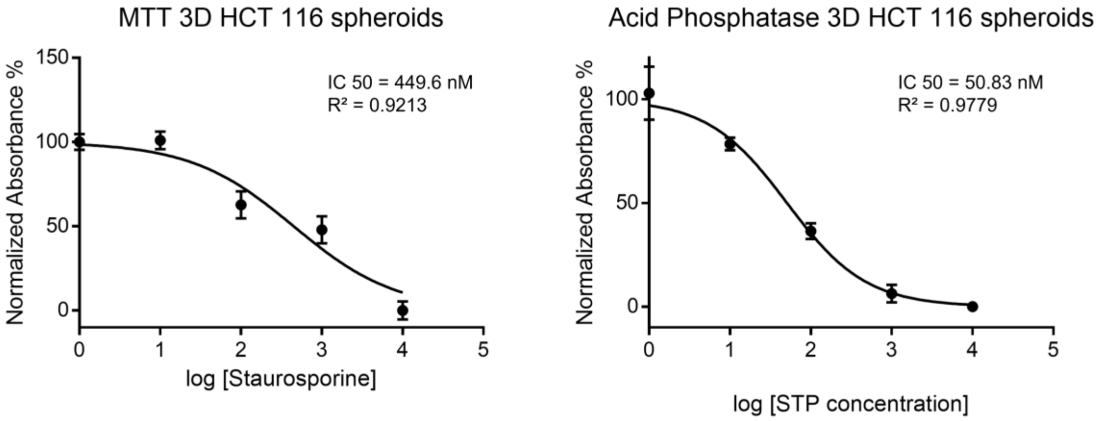

Figure S2: IC50 graphs from the optimization of the MTT and acid phosphatase assays on 3D spheroids of the HCT 116 human colon carcinoma cell line. A range of concentrations, from 0.1 nM to 10 µM, of the anticancer drug staurosporine was used to determine the most sensitive method for evaluating cytotoxicity in cell spheroids.

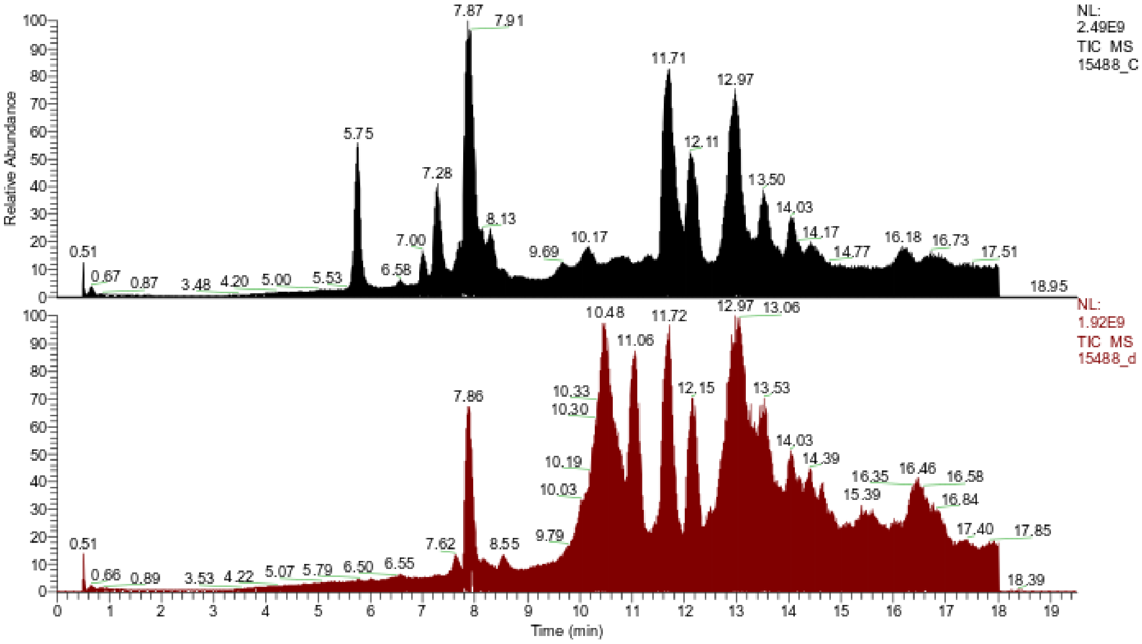

Figure S3. Total ion chromatograms of fractions LEGE 15488_C (upper) and LEGE 15488_D (lower).

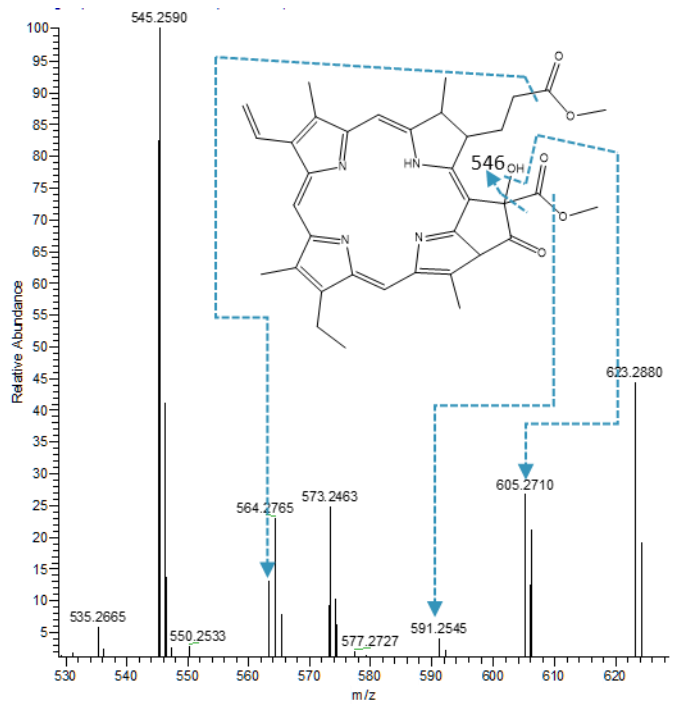

Figure S4. MS/MS spectrum of the protonated molecule at *m/z* 623.2865. The MS2 fragments and molecular formula were consistent with the tentative identification as 13^2-hydroxy-phaeophorbide a methyl ester.

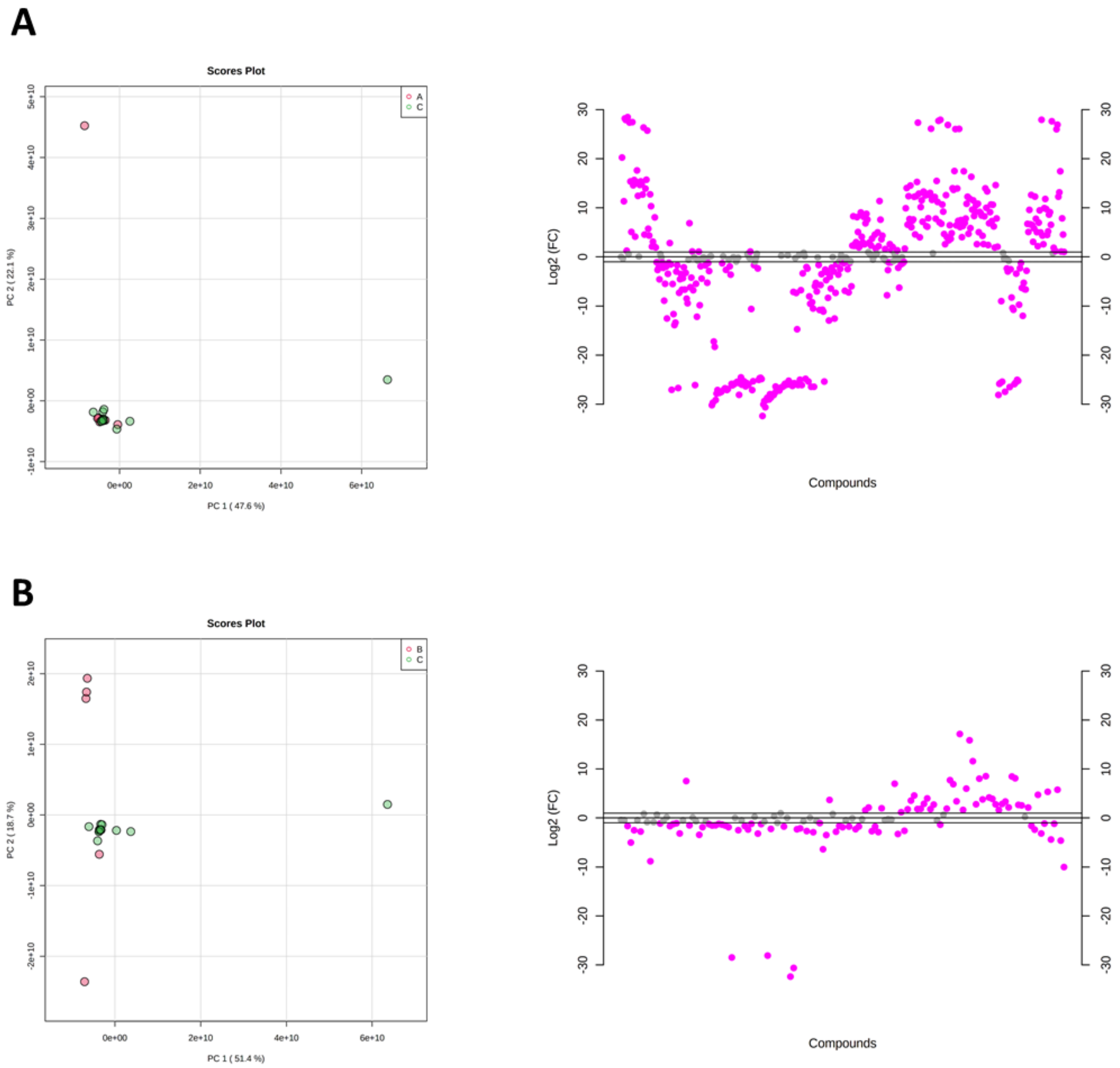

Figure S5. Principal Component Analysis (PCA) and Fold Change plots of the untargeted metabolic analysis of group A/C (A) and group B/C (B).
